# Evaluation of transcriptomic changes after photobiomodulation in spinal cord injury

**DOI:** 10.1038/s41598-025-87300-4

**Published:** 2025-01-25

**Authors:** Andrew R. Stevens, Mohammed Hadis, Hannah Alldrit, Michael R. Milward, Valentina Di Pietro, Deena M. A. Gendoo, Antonio Belli, William Palin, David J. Davies, Zubair Ahmed

**Affiliations:** 1 Neuroscience and Ophthalmology, Department of Inflammation and Ageing, School of Infection, Inflammation and Immunology, College of Medicine and Health, University of Birmingham, Edgbaston, Birmingham, B15 2TT UK; 2 NIHR Surgical Reconstruction and Microbiology Research Centre, University Hospitals Birmingham, Birmingham, B15 2TH UK; 3 Phototherapy Research Group, School of Dentistry, College of Medicine and Health, University of Birmingham, Birmingham, B5 7EG UK; 4 School of Dentistry, College of Medicine and Health, University of Birmingham, Birmingham, B5 7EG UK; 5 Department of Cancer and Genomic Sciences, School of Medical Sciences, College of Medicine and Health, University of Birmingham, Birmingham, B15 2TT UK; 6 Centre for Trauma Sciences Research, University of Birmingham, Edgbaston, Birmingham, B15 2TT UK; 7 Centre for Neurogenetics, University of Birmingham, Edgbaston, Birmingham, B15 2TT UK; 8 Institute for Interdisciplinary Data Science and AI, University of Birmingham, Birmingham, B15 2TT UK

**Keywords:** Photobiomodulation, Spinal cord injury, Low-level laser therapy, Neurotrauma, Neuroprotection, Neuroregeneration, Transcriptomics, Neuroscience, Molecular neuroscience, Regeneration and repair in the nervous system

## Abstract

Spinal cord injury (SCI) is a significant cause of lifelong disability, with no available disease-modifying treatments to promote neuroprotection and axon regeneration after injury. Photobiomodulation (PBM) is a promising therapy which has proven effective at restoring lost function after SCI in pre-clinical models. However, the precise mechanism of action is yet to be determined. Here, we used an in-vivo model of SCI in adult rats that received daily PBM (660 nm, 24 mW/cm^2^, 1 min) and at three days post-injury, the injured spinal cord segment was harvested and subjected to whole transcriptome sequencing and subsequent pathway analysis (generally applicable gene-set enrichment (GAGE)). Pathway analysis demonstrated 1275 differentially expressed genes (DEGs) after PBM treatment, of which 397 were upregulated and 878 were downregulated. Key pathways were significantly enriched, including 8.6-fold enrichment of “neuron projection morphogenesis” (adjusted *p* = 8.10 × 10^− 14^), with upregulation of Notch3, Slit1/Robo2 and Sema3g pathways. Ribosomal and oxidative phosphorylation pathways and NADH dehydrogenase were downregulated, and there was upregulation of ATP-dependent activity, cAMP and calcium signalling pathways. Key genes in apoptotic pathways were downregulated, as were S100 and cyclo-oxygenase components. Together, our study supports the favourable effects of PBM in promoting neuroregeneration and suppressing apoptosis after neurological injury. Further findings from pathway analysis suggest that downregulation of metabolism-associated pathways is a mechanism by which acute post-injury mitochondrial dysfunction may be averted by PBM therapy.

**Supplementary Information:**

The online version contains supplementary material available at 10.1038/s41598-025-87300-4.

## Introduction

It is estimated that more than 909,000 people suffer spinal cord injury (SCI) each year, with each case incurring an average cost of $1.4 million^1,2^. SCI is common in young people, frequently caused by motor vehicle accidents, falls and violence and can lead to permanent disabilities including loss of function, pain, and loss of bladder, bowel and sexual function^3^. These symptoms are often permanent, due to factors such as the low intrinsic capacity of central nervous system (CNS) neurons to regrow after injury and the presence of axon growth inhibitory molecules in both the wound site and the environment of the damaged neuron^3^. These factors prevent regeneration of axons and at present there are no drugs that can promote repair of CNS neuron and restore their lost function^4^. Pharmacological therapies targeting single pathways have shown some promise, but a multitude of pathophysiological mechanisms need to be targeted to promote effective repair^3,5^.

Photobiomodulation (PBM), which uses red or near-infrared (R/NIR) light to promote a therapeutic effect, acts principally on mitochondria, where cytochrome C oxidase is the main photoacceptor in the cell^6,7^. This results in modulation of the mitochondrial membrane potential, reduced levels of reactive oxygen species (ROS) and increased availability of adenosine triphosphate (ATP)^8,9^. Via emerging mechanisms, this initiator process triggers multiple favourable downstream pathways, mitigating apoptosis, neuronal damage, neuroinflammation, and promotes proliferation and neuronal regeneration^8,10,11^. In recent years, interest in PBM has expanded from topical applications to pathologies of deeper anatomical structures, such as the CNS^10–12^. PBM promotes neuronal survival and repair after neurotrauma, particularly in traumatic brain injury (TBI) and SCI alike^10–12^.

In SCI, PBM not only promotes functional recovery and improves histological outcomes in rodent models of SCI, but it also improves other favorable mechanisms in the repair process^12–14^. These include attenuation of macrophage/microglia/astrocyte (M1/A1 phenoytype) polarization^15,16^, neuroinflammation through STAT3 inhibition^14^ and neuronal mitochondrial regulation^17^. We have previously shown that 660 nm PBM at 24.4 mW/cm^2^ delivered daily for seven days improved functional and histological outcomes after SCI, with neuroprotection, neuroregeneration and a reduction of the lesion cavity size and extent of glial scarring^18^.

The specific mechanisms of PBM (specifically R/NIR, 600–850 nm) and its interaction with cells of the CNS may be understood on four levels: (1), primary mechanisms: a basis of photobiological interaction where photonic energy is absorbed, and a biological effect initiated; (2), secondary mechanisms: initiator biological mediators, with their altered activity directly initiated by photon absorption; (3), tertiary mechanisms: subsequent molecular cascades and cellular level responses which occur as a consequence of secondary mechanisms; and (4), quaternary mechanisms: tissue/organ/system level effects, occurring due to events at a molecular and cellular level.

To further the development of PBM-based therapies for CNS injury, an understanding of the mechanisms by which PBM results in favourable effects is required. Such insights may also lead to improvements in the design of PBM parameters to achieve optimal effects. The primary aim of this work therefore was to use a transcriptomic approach to explore the mechanisms of PBM acting at the site of injury after SCI.

## Results

### Differentially expressed genes after PBM

**Fig. 1:**
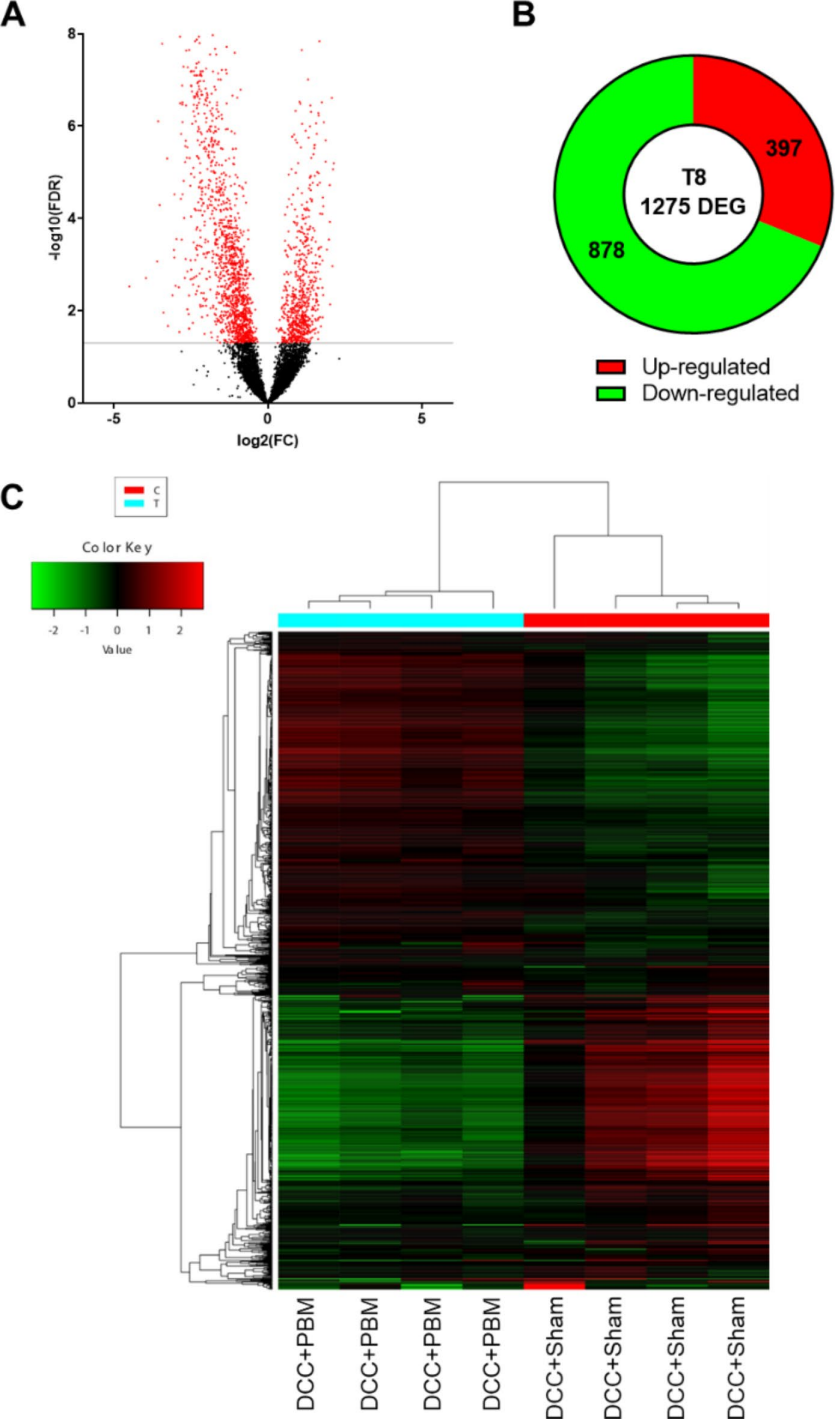
PBM causes changes in differentially expressed genes (DEGs) after SCI. (**A**) Volcano plot with identification (in red) of DEGs defined by FDR < 0.05. (**B**) number of DEGs up or downregulated after PBM in SCI. (C) Heatmap of top 2000 DEGs in DCC + PBM and DCC + Sham (DCC and sham light therapy) groups (*n* = 4 per group). DEGs = differentially expressed genes; FDR = false discovery rate.

After DCC + PBM, we identified 1275 differentially expressed genes (DEGs) with an adjusted p value (FDR) < 0.05 and a fold change greater than two (See Supplementary File 1 and 2 for full lists). There were 397 DEGs that were upregulated and 878 that were downregulated, in response to PBM (Fig. 1A and B). A heatmap of the top 2000 DEGs (based on fold change) showed clear differences between DCC + PBM and DCC + Sham groups, with clustering of DEGs that were highly up/downregulated in DCC + PBM- versus DCC + Sham-treated rats (Fig. 1C).

### Clustering of genes after PBM

**Fig. 2:**
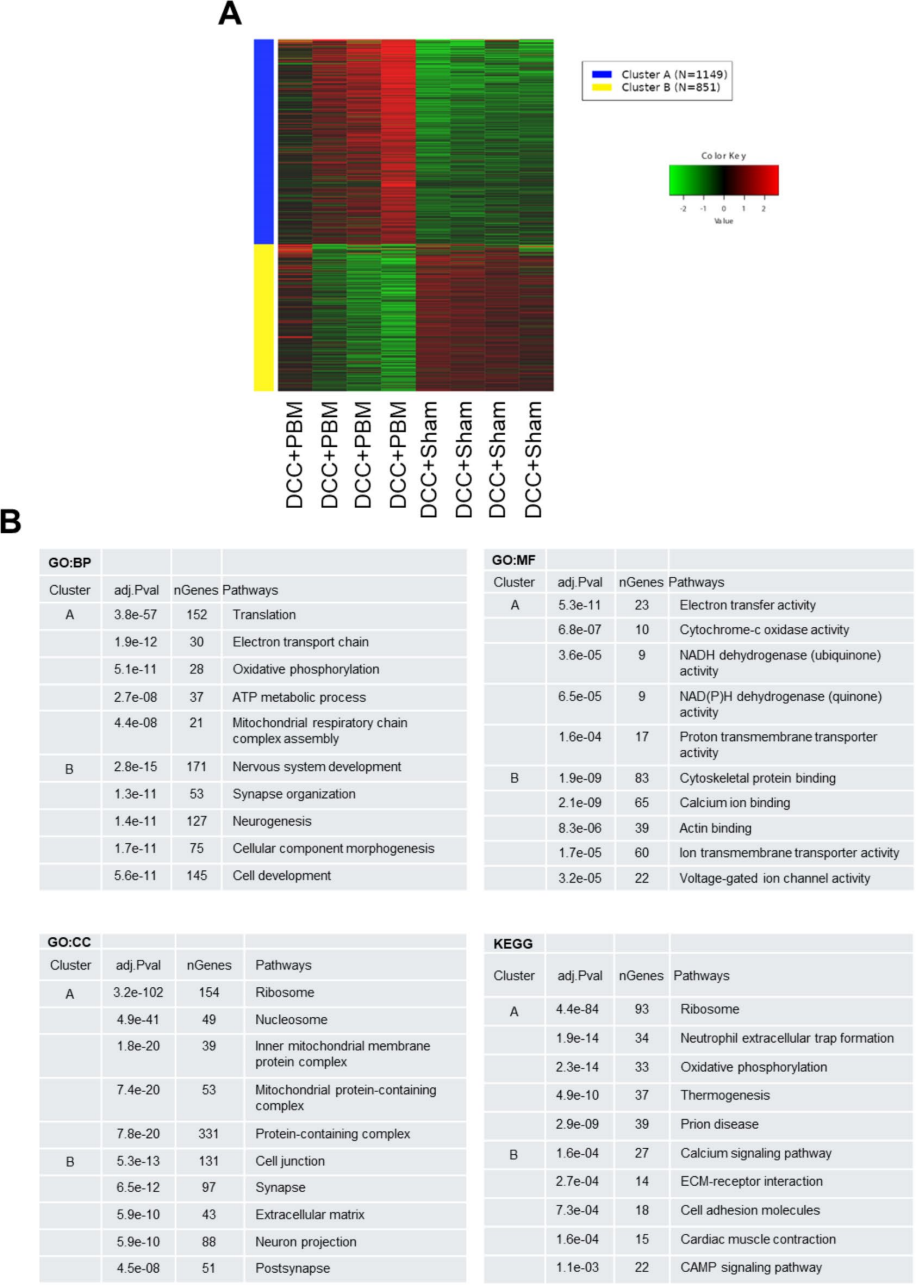
Gene clustering and pathway analysis. (**A**) k-means clusters across DCC + sham and DCC + PBM groups with relative expression per specimen (**B**) GO and KEGG enrichments per cluster. adj.Pval = false discovery rate p value; nGenes = number of genes in pathway; GO = gene ontology; KEGG = Kyoto encyclopaedia of genes and genomes; GO: BP = GO biological processes; GO: MF = GO molecular function; GO: CC = GO cellular component. Permission has been obtained from Kanehisa laboratories for using KEGG pathway database^19^.

To identify clusters of functional activity within DEGs, k-means clustering was performed using the top 1000 DEGs as input for initial GAGE method pathway enrichment across GO and KEGG libraries (Fig. 2A and B). Across all libraries, clear divisions of enriched pathways were identifiable for each cluster. Cluster A was.

predominantly metabolic, with features identified across libraries associated with mitochondrial function, the electron transport chain and ATP synthesis. Cluster B was predominantly associated with pathways related to neuroregeneration, neurite outgrowth, cytoskeleton development and synaptic transmissions.

### Gene ontology and pathway analysis

**Fig. 3:**
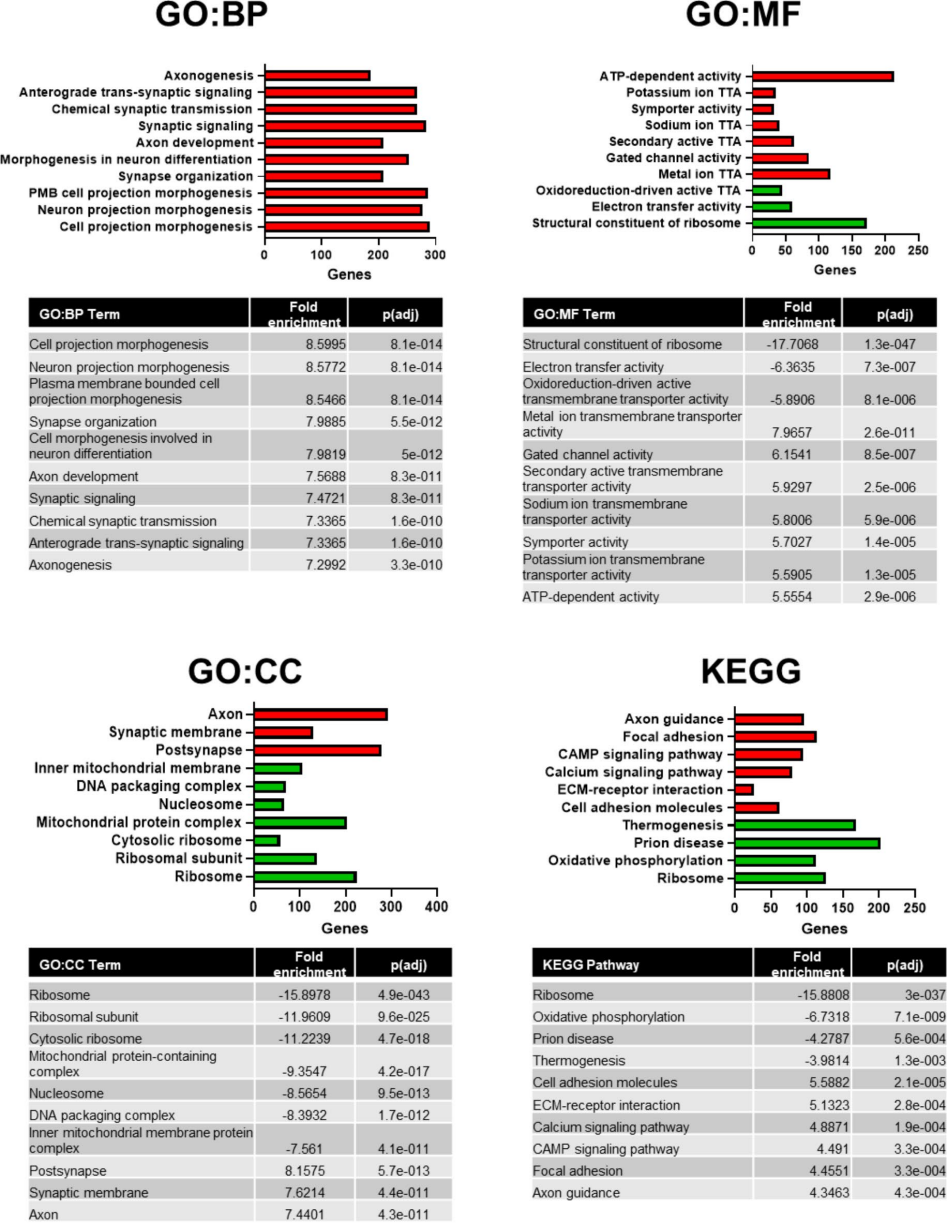
Bar plots with tables demonstrating the top ten relevant hits for each library, with fold enrichment of the pathway. Upregulated pathways shown in red, downregulated pathways shown in green. P(adj) = false discovery rate p value; GO = gene ontology; KEGG = Kyoto encyclopaedia of genes and genomes; GO: BP = GO biological processes; GO: MF = GO molecular function; GO: CC = GO cellular component; TTA = transmembrane transporter activity. Permission has been obtained from Kanehisa laboratories for using KEGG pathway database^19^.

GAGE analysis was performed, using all DEGs, limited to GO and KEGG libraries, in the iDEP.96 web-based bioinformatics platform (https://bioinformatics.sdstate.edu/idep96/). In this analysis, inclusion of fold enrichment of DEGs generated insights on the direction of pathway enrichment. As shown in Fig. 3, upregulated GO terms (in red) include: neuron projection morphogenesis; ATP-dependent activity and synaptic membrane. Downregulated GO terms (in green) included: ribosome; mitochondrial protein-containing complex; and electron transfer activity. The top upregulated KEGG pathways included: axon guidance and calcium signalling pathway. Downregulated KEGG pathways included ribosome and oxidative phosphorylation. These were used to select pathways for network visualisation exploratory analysis.

**Fig. 4:**
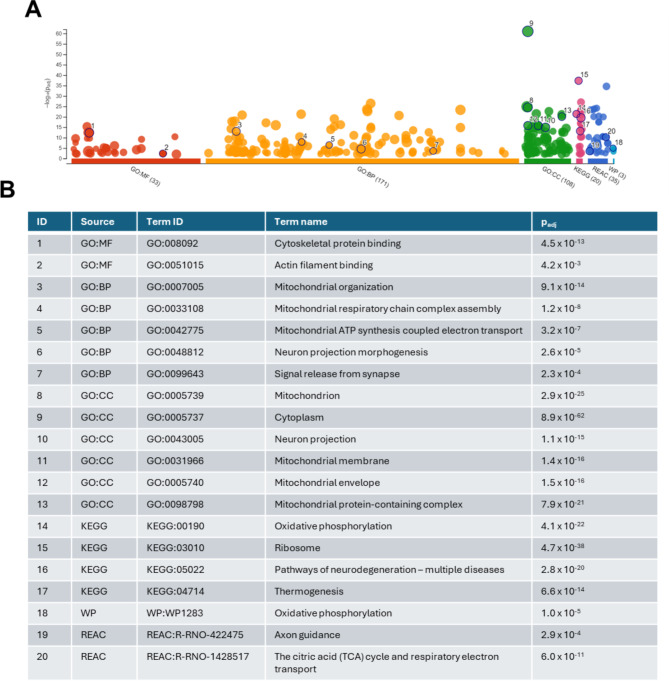
g: profiler interface outputs. (**A**) Top 20 relevant hits highlighted. (**B**) List of top 20 pathway hits across all libraries (GO and KEGG. Padj = false discovery rate p value; GO = gene ontology; KEGG = Kyoto encyclopaedia of genes and genomes; GO: BP = GO biological processes; GO: MF = GO molecular function; GO: CC = GO cellular component; WP = wikipathways; REAC = reactome pathways. Permission has been obtained from Kanehisa laboratories for using KEGG pathway database^19^.

These pathway types were also evaluated in g: profiler, which identified enrichment of a similar range of pathway themes (Fig. 4A and B). Closer inspection of the top 20 pathways also showed significant changes related to mitochondrial pathways and oxidative phosphorylation (Fig. 4B) as well as cytoskeletal elements and axon guidance pathways.

**Fig. 5:**
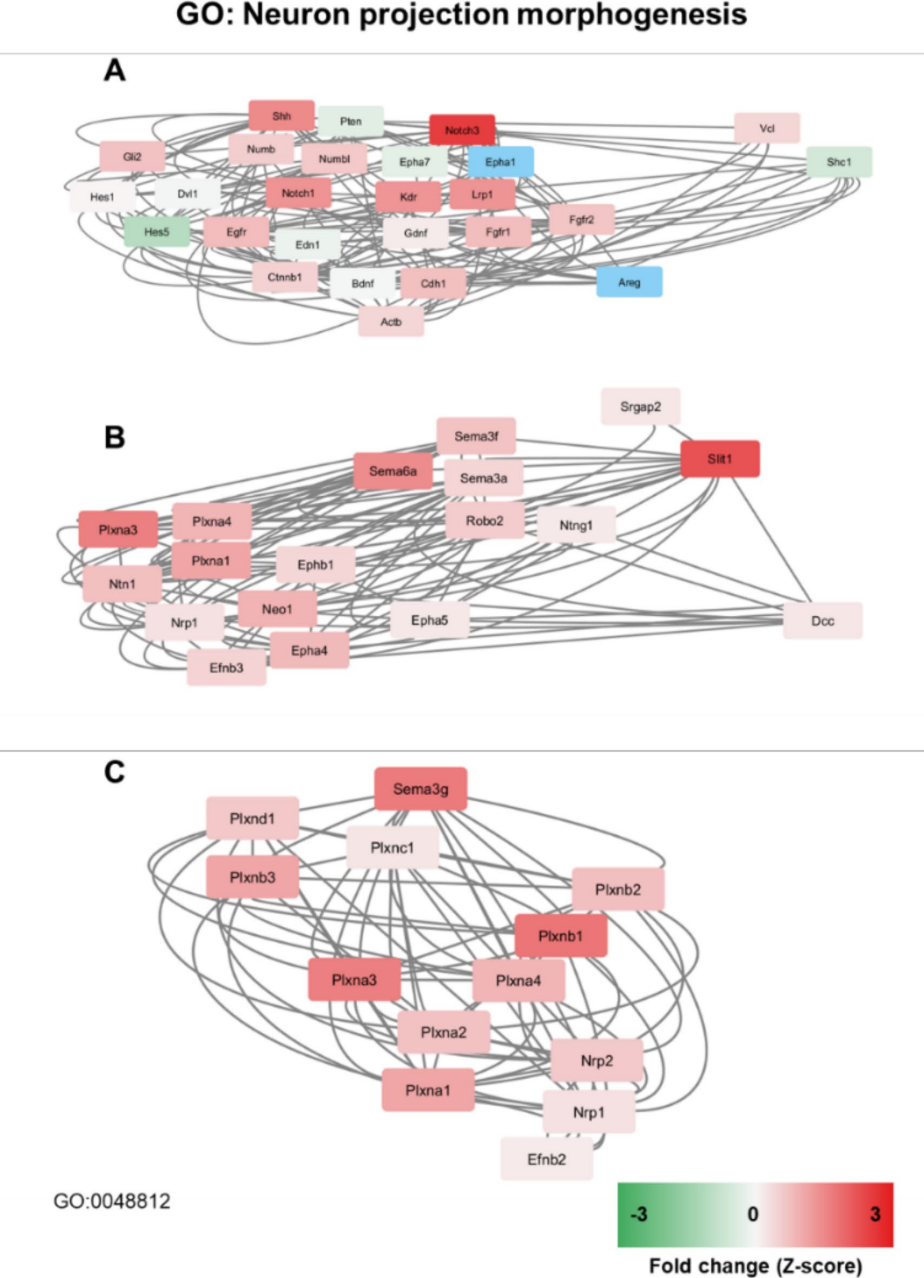
Networks based on top DEGs within neuron projection morphogenesis with their immediate neighbours (undirected). (**A**) Networks based on Notch3. (**B**) Networks based on Robo2 (receptor for Slit1). (**C**) Networks based on Sema3g. GO = gene ontology. Genes shown in blue were filtered from the pathway analysis due to inadequate read counts.

Neuron projection and associated pathways were the predominant theme across many of the pathways identified as enriched. Of these, the GO: BP term “Neuron projection morphogenesis” was identified as a key mechanism in correlation with previous results demonstrating the activity of PBM in promotion of neuroregeneration. Genes from the top ten upregulated DEGs within this pathway were used to visualise interconnected components of this large pathway, based on Notch3 (Fig. 5A), Robo2 (Fig. 5B) and Sema3g (Fig. 5C). This demonstrated key signalling roles for Slit1, Notch1/3, Shh, Sema3/6 and Plxna/b groups.

**Fig. 6:**
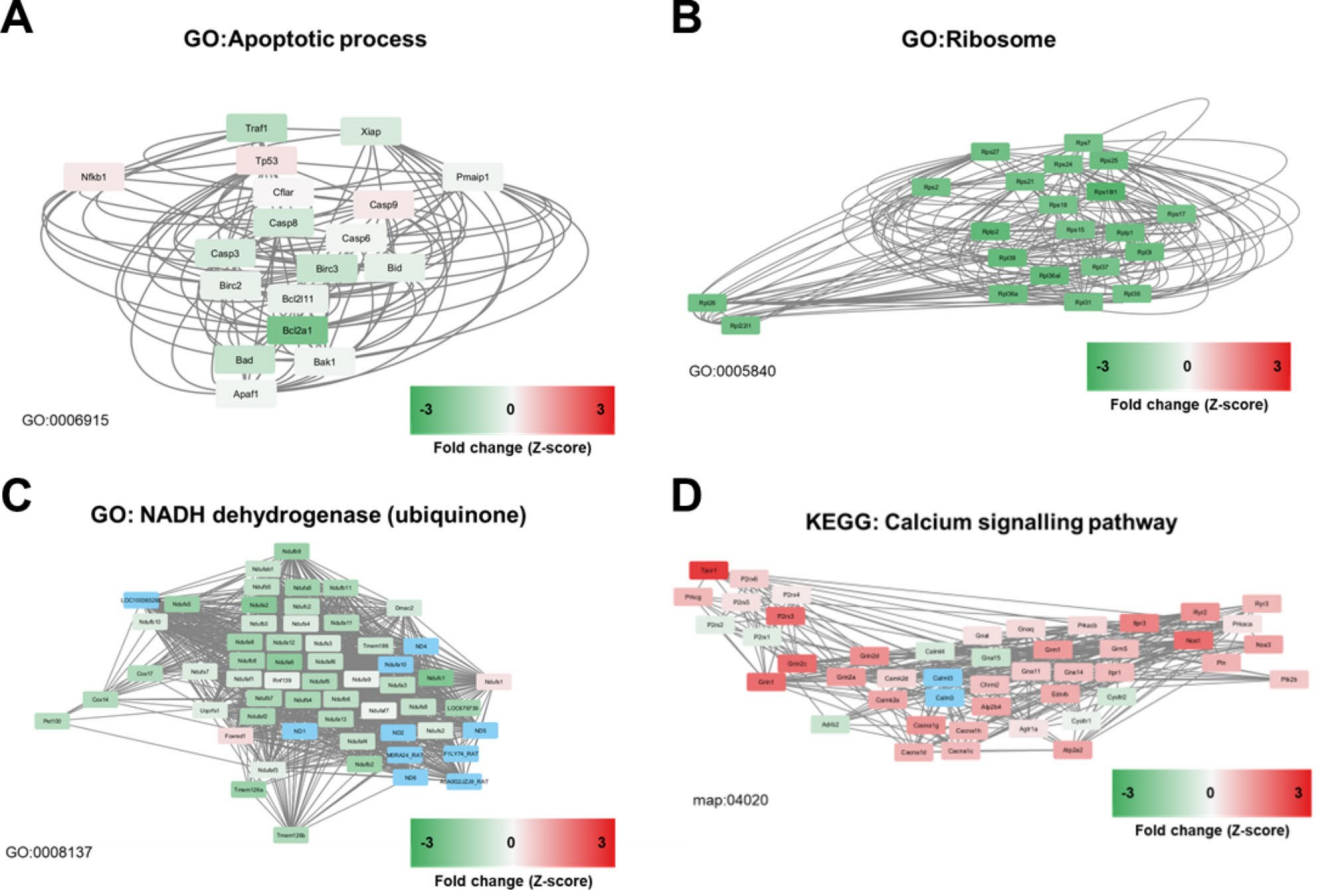
Networks based on top 3 DEGs within selected pathways with their immediate neighbours (undirected). GO = gene ontology. (**A**) apoptotic, (**B**) ribosome, (**C**) NADH dehydrogenase and (**D**) calcium signalling pathways. Genes shown in blue were filtered from the pathway analysis due to inadequate read counts. Permission has been obtained from Kanehisa laboratories for using KEGG pathway database^19^.

Key downregulated pathways were also identified. GO term “Apoptotic process” showed downregulation in transcription of Casp3/8, Bcl2a1 and Traf1 (Fig. 6A). “Ribosome” (and associated pathways e.g. linked to translation and peptide biosynthetic processes) demonstrated negative fold enrichments. The ribosome pathway is shown as a network in Fig. 6B, demonstrating exclusively downregulated transcription of Rps and Rpl family genes contributing to ribosomal function in the PBM treated specimens. Similarly, nicotinamide adenine dinucleotide hydrogen (NADH) (ubiquinone) pathways showed predominant downregulation across the pathway (Fig. 6C), formed principally of components of the electron transport chain including Nduf and Cox subunits. PBM elicited an overall upregulation of calcium signalling in comparison with untreated controls (Fig. 6D) Visualisation of the effects on KEGG pathway of calcium signalling demonstrated a variety of effects of PBM, including upregulation of nitric oxide synthases (Nos1/Nos3), NMDA receptor subunits (Grin1/Grin2c), P2 ATP receptors and substance P receptor (Tacr1) (Fig. 6D).

**Fig. 7:**
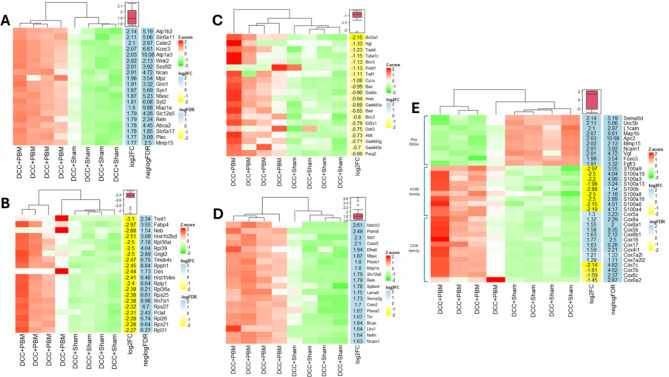
Selected upregulated and downregulated genes after PBM. (**A**) Heat map demonstrating top 20 upregulated genes. (**B**) Heat map demonstrating top 20 downregulated genes. (**C**) Heat map demonstrating top 20 apoptosis associated genes. (**D**) Heat map demonstrating top 20 neuroregeneration associated genes as shown. (**E**) Heat map demonstrating expression of (top) 10 key DEGs (top), S100 genes (middle); and COX family genes (bottom) after PBM treatment. DEGs = differentially expressed genes; FDR = false discovery rate; log2FC = log2 fold change; DCC + Sham = DCC and sham light (control) treatment); DCC + PBM = DCC + PBM treated specimens.

The top 20 upregulated genes after DCC + PBM included: Atp1b2 (ATPase which maintains Na^+^/K^+^ gradients across the plasma membrane); Slc6a11 (terminates inhibitory GABAergic signalling); Celsr2 (cadherin); and Kncn3 (voltage-gated potassium channel (Fig. 7A)). These suggest critical roles for improved regulation of neurotransmitter activity with PBM treatment. Other upregulated genes are associated with neurogenesis, synaptogenesis and cell positioning in neuronal development, including Mpz (myelin protein zero); Syn1 (synapsin 1); Reln; Syt2; Map1a; Slc12a5 and Plec (plectin) (Potokar and Jorgacevski, 2021).

The top 20 downregulated genes after DCC + PBM included: Tnnt1 (a troponin with unknown role in spinal cord injury, upregulated in myelomeningocoele (Murphy et al., 2021)); Fabp4 (fatty acid binding protein); Hist1h2bd (histone); Rpl/Rps (ribosomal protein L/S); Gngt2; Tmsb4x and Pclaf (Fig. 7B). These are predominantly.

associated with transcription and ribosomal activity. The top 20 genes associated with GO: BP term “Apoptotic process” are shown in are shown in Fig. 7C. In addition to those highlighted in Fig. 6A, top downregulated genes include pro-apoptotic genes: Tradd (tumor necrosis factor receptor type 1-associated death domain); and Tuba1c (tubulin alpha 1c). Notably, anti-apoptotic genes Ngf (nerve growth factor) and Birc5 (baculoviral IAP repeat containing 5) were also downregulated.

The top 20 genes associated with GO: BP term “Neuron projection morphogenesis” are shown in Fig. 7D. In addition to those shown in Fig. 5 pathways, this highlights upregulation of: Prdm8 (PR/SET domain 8); Efna5 (ephrin A5); Nfasc (neurofascin) and Lama5 (laminin subunit alpha 5). Figure 7E demonstrates a heat map of key transcriptional changes observed with PBM treatment and highlights further relevant DEGs not represented in pathway analysis elsewhere. This includes: Unc5b (Unc-5 netrin receptor B); L1cam (L1 cell adhesion molecule protein); Vgf (VGF inducible nerve growth factor); Fgfr3 (fibroblast growth factor receptor 3); and Foxo3 (Forkhead box O3). Figure 7E also highlights a uniform downregulation of S100 and COX subunit gene families, which is not represented elsewhere in the pathway analysis but shows consistency across both gene groups and was considered noteworthy.

### Quantitative PCR validation of a selection of up-and down-regulated genes

**Fig. 8:**
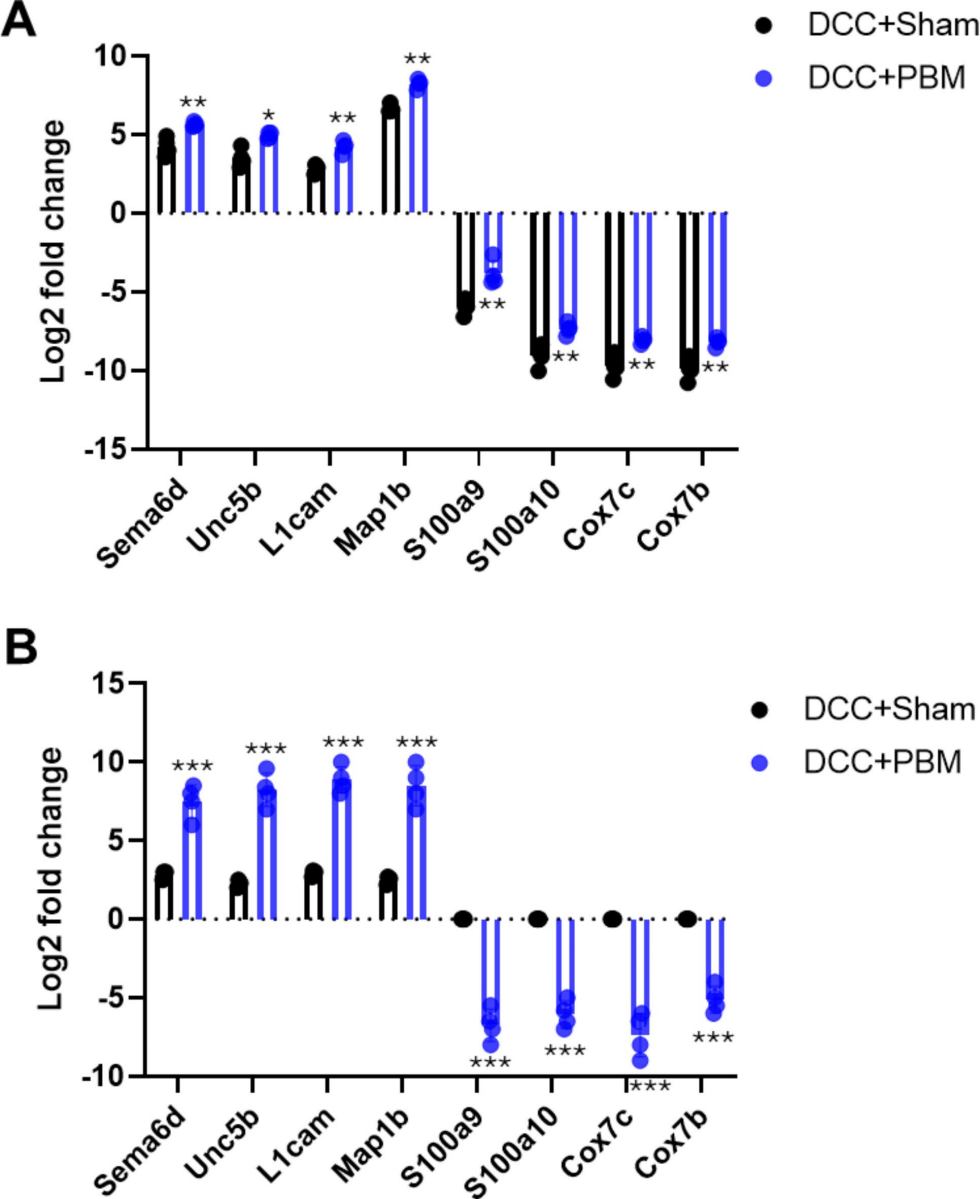
Quantitative PCR (qPCR) validation of selected targets. Selection of differentially regulated gene by (**A**) RNAseq and confirmed using (**B**) qPCR. * = *P* < 0.05, ** = *P* < 0.01, *** = *P* < 0.0001.

Validation of a selection of differentially regulated genes (both up- and down-regulated) demonstrated good agreement between the RNAseq and the qPCR data, confirming that genes such as *Sema6d*, *Unc5b*, *L1cam* and *Map1b* were upregulated between 7-8-fold after DCC + PBM whilst *S100a9*, *S100a10*, *Cox7c* and *Cox7a* were downregulated by PBM between 5-9-fold (Fig. 8A and B).

## Discussion

Using a transcriptomic approach, our study demonstrated acute changes in gene expression elicited by PBM therapy after SCI. Significant enrichment is demonstrated in pathways involved in neural regeneration, synaptogenesis and calcium signalling, accompanied by downregulation of pathways related to mitochondrial turnover and ribosomal activity. Downregulation was also demonstrated in a range of anti-apoptotic genes. This approach has also identified specific transcriptional changes associated with neuroprotection and axonal regeneration in CNS injury, including Mpz, Fabp4 and S100A8/9, which offer new insights into the mechanistic activity of PBM as a therapeutic intervention for improving outcomes after SCI.

### Photobiomodulation and neuroregeneration

The enrichment of pathways associated with neurite outgrowth is an encouraging finding to support previous findings that PBM promoted neuroregeneration after SCI^18^, with upregulation of a range of gene expression profiles, principally centred on Notch3, semaphorins, Slit/Robo and Map1a. This correlates with evidence from broader works demonstrating the synaptogenic activity of PBM after TBI in vivo^20–22^.

Slit1 and its receptor Robo2 have been implicated in promoting neuroregeneration of DRG sensory neurons^23^. Activity of Slit1 after injury has been shown to be regulated by ATP-P2X receptor activity, which correlates with changes shown here^23^. Activity of Notch3 has previously been shown as a key signalling pathway in neuronal differentiation^24^ and also in neurovascular repair after CNS trauma^25^. The upregulation of semaphorin and plexin genes shown here suggests that axonal guidance processes are enhanced. These are key components of neuroplasticity and regeneration^26^, though the neuronal response to this signalling (repulsion vs. attraction) is not clear from this transcriptomic data. The specific functions of these upregulated semaphorins (such as Sema3g) is not well characterized in SCI, though have been associated with plasticity in other parts of the adult CNS^27^.

Synapsin 1 (Syn 1), amongst several other of the upregulated genes described here after PBM treatment, is implicated in modulation of neurotransmitter release, as well as axonogenesis and synaptogenesis^28^. Syn 1 modulates synaptic plasticity by altering the construction of synapses by regulating the reserve pool of synaptic vesicles in a phosphorylation-dependent manner at the presynaptic terminal^29^. As such, the modulation of neuronal excitation through a primary mitochondrial mechanism may therefore be involved in the favourable effects of PBM. Mpz (myelin protein zero) was also shown here to be upregulated in the spinal cord after PBM, though it is known to be specifically expressed in Schwann cells of the peripheral nervous system in mammals^30^. Notably however, Mpz has been implicated in regeneration of the spinal cord in zebrafish and presents a potential novel mechanism for PBM’s neurorestorative effects which warrants further investigation^31^.

### Photobiomodulation and neuroprotection

Transcription of genes associated with cytochrome c mediated apoptosis were demonstrated here to be reduced with PBM application (Diablo, cycs, Bax, caspase-3/8). Identification of favourable effects of PBM on cytochrome c mediated apoptosis provides a link between mitochondrial primary mechanisms (i.e. on the ETC) and its recognised tertiary anti-apoptotic mechanisms^32^. This suggests that mitigation of cytochrome c release from the mitochondria during the acute metabolic distress occurring after SCI may explain these observed benefits. Furthermore, calcium signalling pathways are key regulators of cell death mechanisms^33^. Here we have shown significant alterations in calcium signalling as an effect of PBM: modulating intracellular calcium homeostasis may be a further mechanism by which PBM averts triggering of neuronal apoptosis. Prominent transcriptional changes to neurotransmitter components, as well as ATPase-based ion channels, may be involved in these effects, and this area warrants further investigation.

Beyond these recognised effects of PBM, up and downregulation of key genes after exposure to PBM, as shown here, correlates with a variety of therapeutic targets for spinal cord injury. For example, Fabp4 was strongly downregulated with PBM treatment, and inhibition of this protein has been shown to improve locomotor function in rats after SCI by modulating macrophage and microglial activity^34^. Modulation of the neuroinflammatory response has been consistently recognised as a key function of PBM in facilitating its favourable effects when applied acutely after neurotrauma, and Fabp4 regulation presents a possible mediator for this effect^13–15,17,35–37^.

Linking recognised pathways from transcriptomics to observations such as reduced inflammation, has generated further hypotheses. For example, S100A8/9 complex has a role both in intracellular calcium sensing but is also released in pro-inflammatory states whereupon it stimulates leucocyte migration and inflammatory cytokine release^38^. S100A8/9 has also been implicated in autophagy and apoptosis, and downregulation of expression of these components with PBM treatment may prove a novel mechanism by which PBM reduces neuronal apoptosis in neurotrauma^39^.

### Photobiomodulation and metabolic function

PBM is known to promote metabolic changes in neurons by enhancing neuronal function, as evidenced by restored cytochrome c oxidase and ATP levels^40^. In support of this, we showed GO: MF terms that were enriched included ATP-dependent activity and metal ion transmembrane transporter activity, which support described mechanisms of ATP mediated calcium signalling pathway activation, and activation of calcium homeostatic processes in the ER. This is further supported by activation of calcium signalling pathways (Fig. 6) including P2 receptors. Here there are also marked upregulation of NMDA receptor subunits (e.g. Grin1, Grin2c), which may be a mechanism by which ATP dependent processes activate neurite outgrowth: it has been previously shown that inhibition of activation of NMDA receptors impairs dendritic spine outgrowth, with ER implicated in maintenance of neurite outgrowth^41^. Increases in calcium and cAMP activity have been associated with growth cone morphogenesis^42^ and are recognised effects of PBM^43^. Upregulation of inducible NOS seen in this calcium signalling pathway also correlates with previous findings^44,45^.

### Mechanisms of photobiomodulation

The transcriptomics results have offered some valuable insights into the action of PBM in neurotrauma. Most notably, there are marked similarities between the PBM treated state and a hypometabolic state previously described in more mild TBI in comparison to moderate and severe injuries^46,47^. In this work, Di Pietro and colleagues used a stretch injury model of organotypic hippocampal slice cultures subjected to mild, moderate and severe stretch injury. This was associated with a downregulation in NADH subunits, COX subunits, ATP synthases and histone clusters, in a remarkably similar pattern to that shown here. The authors describe this as a “hibernational state” or “energy conservation programme”, only achievable in mild injury, which mitigates mitochondrial dysfunction and the ensuing oxidative stress patterns and apoptosis.

Administration of PBM then may achieve a similar “minimally energetic” state in the acute post-injury phase, whereby the supportive action of PBM on mitochondrial function acts to allow energy conservation. This may be involved in a reduction in activation of ribosomal stress responses^48^. In combination with averted activation of cytochrome c apoptotic pathways, this state may not only minimise apoptotic processes but permit energy use toward growth cone activation (supported by augmented calcium homeostasis and signalling pathways). These processes may also relate to wider mitochondrial processes: regulation of mitochondrial fission is an emerging mechanism by which PBM is neuroprotective in SCI, with abnormal fission leading to Bax-mediated cellular apoptosis after injury^49^ (Li et al., 2023b). Increased mitochondrial fission is associated with more severe injury in TBI^50^ and regulation of this process may support transition of the severely injured brain and spinal cord into a more “mild” phenotype injurious state.

### Limitations

The transcriptomics analysis has generated supportive evidence for existing hypotheses, as well as generating some novel hypotheses for future research. However, the use of a single set of PBM parameters with a single timepoint post-injury somewhat limits the insights that can be derived from this work. Similarly, no Sham control group was used to demonstrate a relative baseline, which would be of particular interest for comparison of metabolic activity-related transcription with that of the injured-treated condition. Furthermore, the tissue used here was a homogenate of the entire injury site, and as such contains the transcriptome of all cell types within the tissue. As such, resolution of any particular mechanism to within certain cell types (particularly distinguishing between non-neuronal cells) is not possible for intracellular pathways which are well preserved between cell types. Future use of single-cell RNAseq methods may yield more insights into cell-type specific reactions to PBM.

A further limitation of this study could also be that we only surveyed gene changes after 3 days of PBM treatment. We chose this timepoint based on our previous study which showed that 3 days of consecutive PBM treatment maximally activated the axon growth signaling pathway in appropriate dorsal root ganglion neurons^18^. Since the effects of PBM are dose-dependent, this gave us the best chance of determining global gene changes at a timepoint when maximal activation of the pro-regenerative pathway had occurred. However, future studies should include additional timepoints which could determine how sustained the beneficial responses are and whether there are time-dependent changes in this response.

In conclusion, the findings described here have generated important and novel insights into the mechanisms of action of PBM in SCI. The effects of PBM have been elucidated using transcriptomics from an in vivo SCI model, which support a hypothesis that primary effects within the mitochondria enhance intracellular calcium signalling, reduce necessity for mitochondrial component transcription, reduce cytochrome c/calcium mediated-apoptosis and promote neuronal projection development. Such insights are important to support the translation of medical devices using PBM to clinical contexts, by providing a more well-defined rationale for use.

## Methods

### Animals

We used adult 6-8-week-old male Sprague-Dawley rats (190–250 g; Charles River, Margate, UK) for all experiments. Rats were maintained in a 12-hour light/dark cycle with free access to food and water ad libitum. All animal procedures were ethically approved by the University of Birmingham’s Animal Welfare and Ethical Review Board and licensed by the UK Home Office (PP3851114, protocol 4). Surgeries conformed to the guidelines of the UK Animals Scientific Procedures Act, 1986. Power calculations were performed at the outset using the NC3Rs Experimental Design Assistant (EDA) and all animals were randomly assigned to treatment groups and masked to the experimenters throughout and until analysis was complete. The NC3Rs EDA recommended group sizes of *n* = 4. No animals were excluded for any reason and this study conformed to the ARRIVE guidelines for reporting of in vivo experiments. Analgesia was provided pre- and post-injury as standard and as advised by our establishment’s named veterinary surgeon.

### In vivo spinal cord injury

In vivo SCI modelling was performed as described by us previously^51–55^. Briefly, rats were anaesthetised using 5% isofluorane with 1.8 l/min O_2_ and after a partial laminectomy at thoracic level 8 (T8), the dorsal columns (DC) were crushed bilaterally (dorsal column crush injury (termed DCC from herein)) using calibrated watchmaker’s forceps, pre-set to create a lesion 1 mm wide and 1 mm deep, straight through the meninges, creating a reproducible injury in each animal. Animals were treated with either PBM (DCC + PBM) or ambient light (Sham control (DCC + Sham)) within 15 min of performing the injury as described below, before being allowed to recover and returned to their homecages.

### In vivo photobiomodulation

PBM administration was performed as described by us previously^18^. Briefly, a laser source coupled to a diffuser probe was used to deliver 660 nm PBM transcutaneously, using the same treatment parameters. PBM was directed over the lesion site and in line with T8 in the rostro-caudal plane within 15 min post-injury. Subsequent doses were given every 20–24 h, for 3 days whilst animals were awake and using gentle restraint. *n* = 4 animals were used per group.

### Tissue extraction and RNA purification

Instruments and surfaces were prepared with copious amounts of RNase Zap solution (Invitrogen, Massachusetts, USA). Animals were sacrificed at 3 days post-injury by intraperitoneal injection of pentobarbital, and a 0.5 cm section of T8 spinal cord centred on the injury site was isolated and immediately frozen in liquid nitrogen until use^54^. RNA extraction and purification was performed using an RNeasy lipid tissue Mini Kit (Qiagen, Manchester, UK), according to the manufacturer’s instructions. The tissue was removed from liquid nitrogen storage and homogenised in 1 ml QIAzol lysis reagent (Qiagen) using an IKA T10 Ultra-Turbax (IKA-Werke, Staufen, Germany) for 40s per sample. The homogenate was kept at room temperature for 5 min.

200l chloroform was added and the sample was vigorously agitated before a further period of 3 min at room temperature. Samples were centrifuged at 12,000 g for 15 min at 4 °C and the aqueous phase transferred to a fresh tube. 600l 70% ethanol was added and the sample vortexed before duplicate centrifugation through the RNeasy mini spin column for 15s at 8,000 g. The spin column membrane was washed with buffer RW1 by centrifugation for 15s at 8,000 g. Two centrifugation steps were performed at 8,000 g with RPE buffer, first for 15s, then 2 min with fresh RPE buffer. To eliminate carryover, the spin column was centrifuged dry in a fresh collection tube at full speed for 1 min. The RNA was captured by adding RNase-free water to the spin column and collecting the elute. This step was repeated, resulting in a high and low concentration sample. Concentration of RNA was determined using a nanophotometer (N60, IMPLEN, Germany) to derive A260/A280 ratio.

### WT-seq and analysis

Further quality control measures and library preparation for whole transcriptome analysis were outsourced to Qiagen (Germany). RNA integrity (RIN) of > 7 were confirmed for each sample prior to library preparation on an Agilent Bioanalyser and DNAse and gDNA contamination was removed as standard by Qiagen before library preparation. Library preparation was performed using the QIAseq Stranded Total RNA Library Kit with QIAseq FastSelect rRNA and globin depletion. QIAseq FastSelect rRNA HMR was used to reduce the amount of unwanted RNA species. After first and second strand synthesis, the cDNA was end-repaired and 3’ adenylated. Sequencing adapters were ligated to the overhangs. Adapted molecules were enriched using 16 cycles of PCR and purified by a bead-based cleanup. Library preparation was quality controlled using capillary electrophoresis (High Sensitivity Tape D1000) and high-quality libraries were pooled based on equimolar concentrations. The library pool(s) were quantified using qPCR and optimal concentration of the library pool used to generate the clusters on the surface of a flowcell before sequencing on a NovaSeq (Illumina Inc., Madison, USA) instrument (2 × 75, 2 × 10) according to the manufacturer instructions (Illumina Inc.). Raw data was de-multiplexed and FASTQ files for each sample were generated using the bcl2fastq software v2.20.0.422 (Illumina inc.).

Trimmed raw read counts were analysed using iDEP96^56^ (http://bioinformatics.sdstate.edu/idep96/). Genes were pre-processed to limit genes to 0.5 counts per million and appearing in at least one library. Samples were grouped into PBM treatment (DCC + PBM) and Sham light treatment (DCC + Sham) for comparative analysis (*n* = 4 per group). Count data was transformed for clustering and principal component analysis using EdgeR: log2(CPM + 1). Differentially expressed genes (DEGs) were identified using DESeq2 with an FDR adjusted p value of 0.05, calculated using the Benjamini-Hochberg method, specifying a minimum fold change of 2. Volcano plots were rendered using GraphPad Prism (v10.2.1 GraphPad, La Jolla, CA, USA). k-means analysis was performed in iDEP96 using the top 1000 variable genes (with false discovery rate (FDR) < 0.05) limiting clusters to 2. Heatmaps were plotted in R^57^ (v4.3.3 www.R-project.org) using ggplot2^58^ (v3.5.1) (for top 2000 variable genes) and using the ComplexHeatMap package^59^ (v2.20.0) (for top 10/20 up/downregulated genes and specific gene families).

Pathway analysis using generally applicable gene-set enrichment (GAGE) method was performed with a pathway significance cut off of 0.05 (FDR). Gene set size range used was 15–500. The top 20 pathways for gene ontology (GO) terms and Kyoto encyclopedia of genes and genomes (KEGG) terms were identified^19,60–62^. For KEGG pathway analysis, absolute fold changes were used. Supplementary functional enrichment analysis was performed in g: profiler^63^, selecting the top 20 relevant hits across all databases. For exploratory network analysis of GO terms and KEGG pathways, pathway networks were retrieved from STRING Database^64^ (v12) and imported into Cytoscape (v3.10.1, Washington, USA). For large pathways, central nodes were selected as the hub from the top three hits (by fold change, filtered by FDR < 0.05). First neighbours of selected nodes were integrated into a new pathway (undirected neighbours as identified by Cytoscape). Fold changes were imported from iDEP96 as Z-scores and mapped to String network components using integrated functionality of Cytoscape. Z-scores were used to display fold change of nodes.

### Quantitative polymerase chain reaction (qPCR)

A selection of the 10 key differentially expressed genes were validated by qPCR using an independent set of samples with DCC + Sham and DCC + PBM treatment (*n* = 4/group). Using the RNA prepared as described above, cDNA was synthesized using SuperScript III (ThermoFisher Scientific) and qPCR was performed using TaqMan gene expression assays and validated PCR primers (Supplementary Table 1), according to the manufacturer’s instructions (ThermoFisher Scientific). Target gene expression was normalized to the housekeeping genes, *Gapdh*, using the delta threshold cycle (ΔΔCt) method. Relative gene changes were calculated using the equation: relative gene changes = 2^−ΔΔCT^ where ΔCt = ΔCt_target_-ΔCt_Gapdh_ and ΔCt_DCC+PBM_-ΔCt_DCC+Sham_.

### Statistical analysis for qPCR

All data are expressed as mean + SD. Statistical analysis was performed using Prism software (version 8.4.3). Relative gene expression determined by qPCR was compared between DCC + Sham and DCC + PBM groups using a two-way ANOVA with Holm’s Sidak multiple comparisons correction. *P* < 0.05 was considered statistically significant.

## Electronic supplementary material

Below is the link to the electronic supplementary material.

Supplementary Material 1

Supplementary Material 2

Supplementary Material 3

## Data Availability

The datasets generated and/or analysed during the current study are available in the ArrayExpress repository, accession number: E-MTAB-14593 (https://www.ebi.ac.uk/biostudies/arrayexpress/studies/E-MTAB-14593). Raw read counts (Supplementary file 1) and differential gene expression (Supplementary file 2) are available as supplementary materials. All datasets supporting this study are also available freely on Figshare.com at 10.6084/m9.figshare.25974742.v1.
