# Supplementary material for: Evaluation of transcriptomic changes after photobiomodulation in spinal cord injury
Source: Sci Rep. 2025 Jan 25;15:3193. doi: 10.1038/s41598-025-87300-4 (PMC11762322; doi:10.1038/s41598-025-87300-4)
Supplement: Supplementary file 3 — Supplementary Material 3 [file 41598_2025_87300_MOESM3_ESM.docx]

**Supplementary Materials**

**Evaluation of transcriptomic changes after photobiomodulation in spinal cord injury**

Andrew R. Stevens^1,2,3^, Mohammed Hadis^3,4^, Hannah Alldrit^5^, Michael R. Milward^4,5^, Valentina Di Pietro^1,2,6,7^, Deena M.A. Gendoo^5,8^, Antonio Belli^1,2,6^, William Palin^3,4^, David J. Davies^1,2,3,6^, Zubair Ahmed^1,2,6,7,^*

^1^Neuroscience and Ophthalmology, Department of Inflammation and Ageing, School of Infection, Inflammation and Immunology, College of Medicine and Health, University of Birmingham, Edgbaston, Birmingham, B15 2TT, UK

^2^NIHR Surgical Reconstruction and Microbiology Research Centre, University Hospitals Birmingham, Birmingham, B15 2TH, UK

^3^Phototherapy Research Group, School of Dentistry, College of Medicine and Health, University of Birmingham, Birmingham UK

^4^School of Dentistry, College of Medicine and Health, University of Birmingham, Birmingham UK

^5^Department of Cancer and Genomic Sciences, School of Medical Sciences, College of Medicine and Health, University of Birmingham, Birmingham UK

^6^Centre for Trauma Sciences Research, University of Birmingham, Edgbaston, Birmingham, UK

^7^Centre for Neurogenetics, University of Birmingham, Edgbaston, Birmingham, B15 2TT, UK

^8^Institute for Interdisciplinary Data Science and AI, University of Birmingham, Birmingham, UK

**Table S1.** Validated PCR primers for selected differentially expressed genes (upregulated = red; downregulated = green) after PBM treatment.

| **Gene** | **Cat no.** | **Assay ID** |
| --- | --- | --- |
| Sema6d | 4351372 | Rn01485136_g1 |
| Unc5b | 4331182 | Rn00573551_m1 |
| L1cam | 4331182 | Rn00493049_m1 |
| Map1b | 4331182 | Rn01494211_m1 |
| S100a9 | 4331182 | Rn00585879_m1 |
| S100a10 | 4331182 | Rn06378613_s1 |
| Cox7c | 4331182 | Rn01493939_g1 |
| Cox7b | 4351372 | Rn00822088_g1 |
| Housekeeping gene | | |
| Gapdh | 4331182 | Rn01775763_g1 |
